# Redesigned and reversed: architectural and functional oddities of the trypanosomal ATP synthase

**DOI:** 10.1017/S0031182021000202

**Published:** 2021-09

**Authors:** Ondřej Gahura, Carolina Hierro-Yap, Alena Zíková

**Affiliations:** 1 Biology Centre, Czech Academy of Sciences, Branišovská 31, České Budějovice, 37005, Czech Republic; 2 Faculty of Science, University of South Bohemia, Branišovská 31, České Budějovice, 37005, Czech Republic

**Keywords:** ATP synthase, cryo-EM, mitochondria, mitochondrial membrane potential, oxidative phosphorylation

## Abstract

Mitochondrial F-type adenosine triphosphate (ATP) synthases are commonly introduced as highly conserved membrane-embedded rotary machines generating the majority of cellular ATP. This simplified view neglects recently revealed striking compositional diversity of the enzyme and the fact that in specific life stages of some parasites, the physiological role of the enzyme is to maintain the mitochondrial membrane potential at the expense of ATP rather than to produce ATP. In addition, mitochondrial ATP synthases contribute indirectly to the organelle's other functions because they belong to major determinants of submitochondrial morphology. Here, we review current knowledge about the trypanosomal ATP synthase composition and architecture in the context of recent advances in the structural characterization of counterpart enzymes from several eukaryotic supergroups. We also discuss the physiological function of mitochondrial ATP synthases in three trypanosomatid parasites, *Trypanosoma cruzi*, *Trypanosoma brucei* and *Leishmania*, with a focus on their disease-causing life cycle stages. We highlight the reversed proton-pumping role of the ATP synthase in the *T. brucei* bloodstream form, the enzyme's potential link to the regulation of parasite's glycolysis and its role in generating mitochondrial membrane potential in the absence of mitochondrial DNA.

## Graphical abstract

**Figure:**
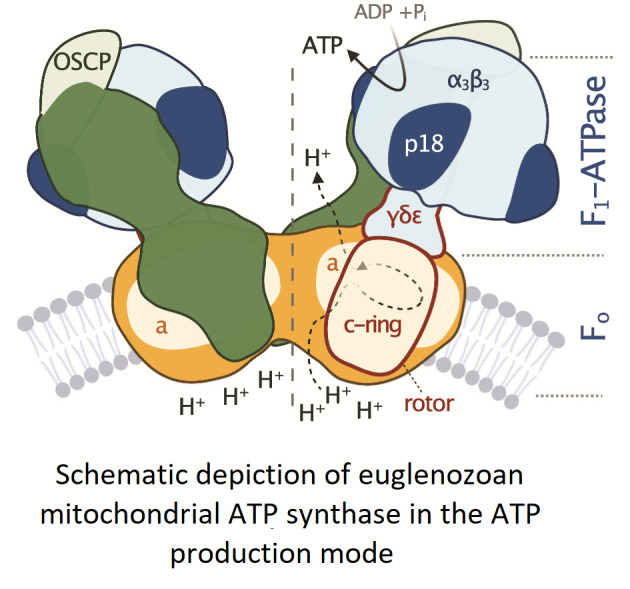

## Introduction

F-type adenosine triphosphate (ATP) synthases (also called F-ATPases of F_1_F_0_-ATPases) are bidirectional turbine-like enzymes coupling ATP synthesis or hydrolysis with proton translocation through biological membranes in bacteria and their endosymbiotic descendants, mitochondria and chloroplasts (for recent reviews see Junge and Nelson, 2015; Walker, 2017; Kuhlbrandt, 2019). When operating in the forward mode, ATP synthases utilize the proton motive force to produce ATP and thus represent fundamental constituents of the oxidative phosphorylation pathway. In the reverse mode, they act as ATP-consuming proton pumps contributing to the generation of electrochemical membrane potential. Bacteria employ both modes depending on the species and growth conditions (Cotter and Hill, 2003). By contrast, mitochondrial ATP synthases in most aerobic eukaryotes function as a major source of cellular ATP and the reversal of their activity is a manifestation of pathophysiological conditions (Campanella *et al*., 2009). Unlike the monomeric bacterial and chloroplastic counterparts, all mitochondrial ATP synthases characterized so far occur in dimers (Arnold *et al*., 1998; Dudkina *et al*., 2005). The wide phylogenetic distribution of the ATP synthase dimers suggests that the enzyme's dimerization is a common feature of all aerobic eukaryotes with oxidative phosphorylation and was possibly present already in the last eukaryotic common ancestor. Dimerization of ATP synthases induces curvature of the inner mitochondrial membrane (Dudkina *et al*., 2006; Davies *et al*., 2012) and the intrinsic propensity of dimers to self-assemble into rows facilitates membrane shaping and governs cristae formation (Anselmi *et al*., 2018; Blum *et al*., 2019). Spatial separation of ATP synthases and electron transport chain (ETC) complexes on rims and flat regions of cristae, respectively, creates a local proton concentration gradient in cristae lumen, enhancing oxidative phosphorylation (Davies *et al*., 2011). Therefore, apart from the enzymatic function, ATP synthases affect mitochondrial physiology by aiding in the determination of submitochondrial ultrastructure.

In *Trypanosoma brucei*, a medically relevant digenetic parasite, the mitochondrial ATP synthase exhibits a 2-fold deviation from its counterparts from traditional model organisms. First, its architecture differs markedly from canonical ATP synthases. Second, its role switches during the parasite's life cycle, from being an ATP producer in the insect form to an ATP consumer maintaining the vital mitochondrial membrane potential in the mammalian bloodstream form (BSF). This switch is associated with major rearrangement of mitochondrial morphology, possibly involving altered ATP synthase oligomerization. Unlike in *T. brucei*, in *Trypanosoma cruzi* and *Leishmania*, ATP synthase acts true to its name and participates in the oxidative phosphorylation providing ATP in all life forms of the parasite. In the first part of this review, we discuss the recent progress in our understanding of ATP synthase architecture based on structural characterization of enzymes from various eukaryotic supergroups with a focus on trypanosomal ATP synthase complex. In the second part, we summarize our knowledge of the ATP synthase importance for the disease-causing forms of medically relevant trypanosomatid parasites.

## Unexpected diversity of mitochondrial ATP synthases

**Fig. 1.**
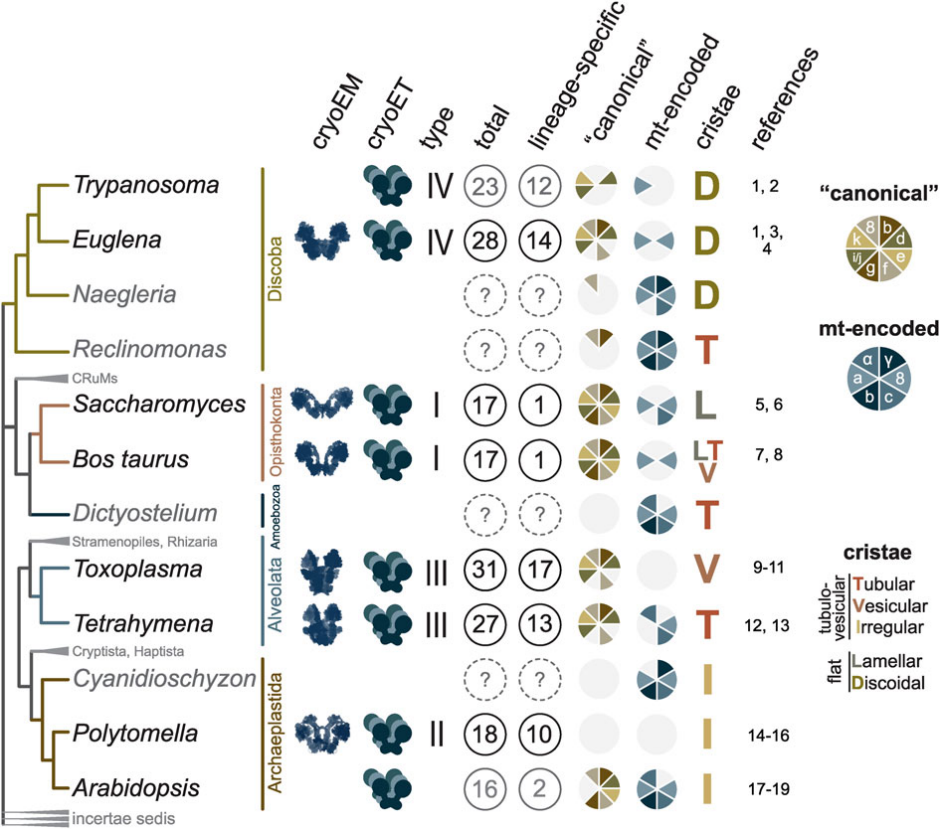
Structural diversity of mitochondrial ATP synthases mapped on the phylogenetic tree of eukaryotes. The figure summarizes structural and proteomic studies of mitochondrial ATP synthases. The phylogenetic tree is based on Burki *et al*. (2020). Organisms with ATP synthases with atomic models obtained by single particle cryo-EM, visualized by cryo-ET (see first two columns) or characterized by MS, and several relative species were included. Major groups without experimental data are shown in small font. Numbers of subunits showed in grey are based on proteomic characterization of purified complexes without available single particle cryo-EM analysis and might be revised in future. ‘Canonical’ subunits are proteins originally identified in opisthokonts, which have divergent homologs in other lineages. Categorization of ATP synthases in types I to IV is based on Kuhlbrandt (2019). Cristae morphology is adopted from Panek *et al*. (2020). The numbered references are following: ^1^ (Muhleip *et al*., 2017), ^2^ (Zikova *et al*., 2009), ^3^ (Muhleip *et al*., 2019), ^4^ (Sathish Yadav *et al*., 2017), ^5^ (Davies *et al*., 2012), ^6^ (Guo *et al*., 2017), ^7^ (Davies *et al*., 2011), ^8^ (Spikes *et al*., 2020), ^9^ (Mühleip *et al*., 2021), ^10^ (Salunke *et al*., 2018), ^11^ (Huet *et al*., 2018), ^12^ (Flygaard *et al*., 2020), ^13^ (Muhleip *et al*., 2016), ^14^ (Blum *et al*., 2019), ^15^ (Murphy *et al*., 2019), ^16^ (Vazquez-Acevedo *et al*., 2006), ^17^ (Klodmann *et al*., 2011), ^18^ (Bultema *et al*., 2009), ^19^ (Senkler *et al*., 2017).

Structural studies of mitochondrial ATP synthases have been key for the understanding of molecular mechanisms of ATP synthesis. The seminal research on the structure of the matrix-facing soluble F_1_-ATPase subcomplex isolated from the bovine heart (Abrahams *et al*., 1994) initiated two decades of dominance of X-ray crystallography in the field. Numerous crystal structures of subcomplexes of bovine and yeast ATP synthases provided atomic models of the enzyme's rotor and the extrinsic part of the peripheral stalk, and revealed the mechanism of the conversion of the rotational force into the cyclic catalysis of ATP formation (reviewed in Junge and Nelson, 2015; Walker, 2017). Due to the striking similarity of the blueprints for bacterial and opisthokontal mitochondrial F-type ATP synthases and their fundamental role in cellular energetics, it was assumed that the mitochondrial enzymes do not differ substantially between eukaryotic lineages. However, only the advance of cryo-electron microscopy (cryo-EM) techniques in the last few years allowed researchers to build atomic models of complete mitochondrial ATP synthases not only from conventional model organisms [mammals (Gu *et al*., 2019; Pinke *et al*., 2020; Spikes *et al*., 2020) and *Saccharomyces cerevisiae* (Guo *et al*., 2017)], but also from protists representing other eukaryotic phyla [*Polytomella* (Murphy *et al*., 2019), *Euglena* (Muhleip *et al*., 2019), *Tetrahymena* (Flygaard *et al*., 2020) and *Toxoplasma* (Mühleip *et al*., 2021)]. From the mechanistic point of view, structures revealed by cryo-EM elucidated how the proton motive force across biological membranes is converted into the torque of the enzyme's rotor (Klusch *et al*., 2017; Hahn *et al*., 2018) and how the symmetry mismatch of the rotor and catalytic subcomplexes is compensated by the flexibility of the peripheral stalk (Sobti *et al*., 2019). From the evolutionary perspective, the structures demonstrated that although the functional core is universally conserved in all F-type ATP synthases, peripheral parts and the dimerization interface of mitochondrial ATP synthases have diverged substantially among eukaryotic lineages. Notably, cryo-EM structures not only allowed identification of novel lineage-specific components, but in some species also revealed conserved subunits, which had not been previously identified by traditional homology searches using known genomes and proteomes (Fig. 1).

**Fig. 2.**
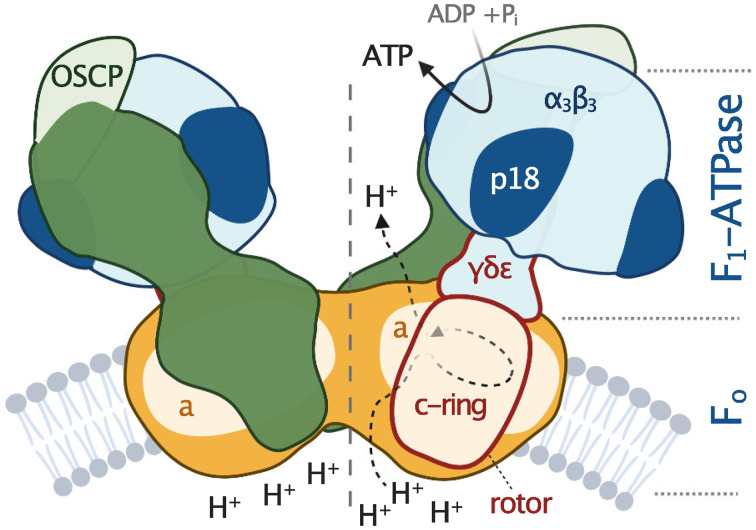
Schematic depiction of euglenozoan mitochondrial ATP synthase in the ATP production mode. The F_1_-ATPase, peripheral stalk and membrane-embedded part are shown in shades of blue, green and orange, respectively. Subunits identified in all reported mitochondrial ATP synthases are pale. The dark green and dark orange regions are composed of conserved and lineage-specific subunits. The p18 subunit is restricted to Euglenozoa. The rotor is outlined in red.

All F-type ATP synthases are composed of two subcomplexes, the membrane-embedded F_0_ and the soluble F_1_, connected by a rotary central stalk and a stationary peripheral stalk (Fig. 2). The globular F_1_ subcomplex, also referred to as F_1_-ATPase, is a pseudo-symmetrical assembly of three heterodimers of *α*- and *β*-subunits organized around single *γ*-subunit, the main constituent of the central stalk. The functional core of F_0_ consists of the a-subunit and a ring of several identical c-subunits (c-ring), providing together a path for protons across the membrane. Proton translocation drives the rotation of the c-ring and the tightly attached central stalk. The torque of the asymmetric *γ*-subunit induces cyclic conformational changes of three active sites on the interfaces of *α*- and *β*-subunits, resulting in the stepwise binding of ADP and phosphate, their condensation into ATP, and its release, emptying the nucleotide binding site for the next catalytic cycle (Abrahams *et al*., 1994). The (*αβ*)_3_-hexamer is prevented from rotation by interaction with the oligomycin sensitivity-conferring protein (OSCP), the uppermost constituent of the extrinsic part of the peripheral stalk.

The entire F_1_-ATPase consisting of subunits *α*_3_, *β*_3_, *γ*, *δ* and *ɛ* (stoichiometry of subunits indicated by the subscripts), OSCP and both proton translocating components (a-subunit and c-ring) are present in all reported mitochondrial ATP synthases. The rest of the enzyme is markedly less conserved (Fig. 2). The highest diversity is observed in the soluble part of the peripheral stalk and in the lumenal and peripheral F_0_ regions, often composed of lineage-specific subunits or lineage-specific extensions of conserved proteins. Subunits involved in the anchoring of the peripheral stalk to the membrane and in clamping the a-subunit to the c-ring (b, d, e, f, g, i/j, k and 8), originally identified in opisthokonts, appear to be conserved in several other eukaryotic groups (Fig. 1). In many cases, their proposed homology is based largely on tertiary and quaternary structure resemblances revealed by cryo-EM. Low sequence similarity precludes identification of the respective homologs in lineages without structurally characterized ATP synthases. Although dimerization is a general hallmark of all reported mitochondrial ATP synthases, subunits constituting the dimer interface differ between eukaryotic lineages, likely due to divergent evolution. Consequently, the shape of dimers and topography of oligomeric assemblies vary in the characterized enzymes, which is reflected in diverse cristae morphology (Fig. 1; Dudkina *et al*., 2010; Davies *et al*., 2012; Muhleip *et al*., 2016; Muhleip *et al*., 2017; Mühleip *et al*., 2021).

## Unique structural features of ATP synthases in Trypanosomatida

**Fig. 3.**
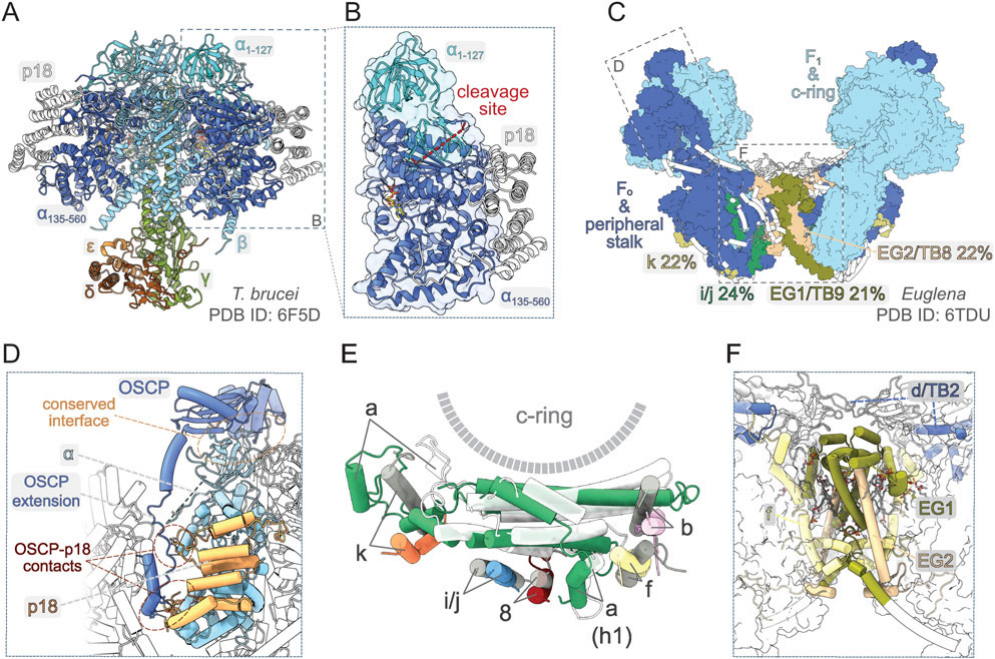
Structural traits of euglenozoan mitochondrial ATP synthases. (A) Structure of F_1_-ATPase from *Trypanosoma brucei* determined by X-ray crystallography (Montgomery *et al*., 2018). (B) The *α*-subunit in euglenozoans is split by proteolytic cleavage at two sites. The euglenozoan-specific subunit p18 associates with the C-terminal fragment *α*_135–560_ and does not contact any other F_1_ component. (C) Structure of the ATP synthase dimer from *Euglena gracilis* (Muhleip *et al*., 2019). Elements with and without homology in *T. brucei* are shown in surface and white cartoon representation, respectively. F_1_-ATPase and c-ring are in pale blue. Subunits with newly proposed homology between *Euglena* and *Trypanosoma* are coloured individually and labelled with names used in both species (Perez *et al*., 2014; Muhleip *et al*., 2019). The sequence identities of respective homolog pairs are shown. All other peripheral stalk and F_0_ subunits are in dark blue. (D) Interaction of the peripheral stalk with F_1_-ATPase. The canonical interaction interface and lineage-specific contacts between OSCP and p18 are shown. (E) Superposition of the a-subunit and adjacent conserved transmembrane helices from *E. gracilis* (subunits coloured individually) and bovine [a-subunit white, all other subunits in grey; PDB ID 6ZPO (Spikes *et al*., 2020)] ATP synthase. (F) Dimer interface of the ATP synthase in *E. gracilis*. Subunits involved in inter-monomer contacts are shown in cartoon representation. Regions that are present in *T. brucei* counterparts based on homology modelling with Swiss model (Waterhouse *et al*., 2018) are coloured individually and *Euglena*-specific elements are in white. All other subunits are shown as transparent surfaces. Ordered lipids (cardiolipins and phosphatidylcholines or phosphatidylethanolamines) localizing to the dimer interface are shown as sticks.

The knowledge about the architecture of mitochondrial ATP synthases in Trypanosomatida, a group of protozoan parasites with monoxenous or dixenous life cycles, is based mostly on studies performed with cultured insect trypomastigotes of *T. brucei*. The composition of the mitochondrial ATP synthase in these cells was determined by mass spectrometry (MS) identification of proteins co-immunoprecipitated with F_1_ and tandem affinity purified using TAP-tagged conserved or newly identified subunits as baits. These biochemical analyses complemented by a comprehensive genome search revealed 23 subunits (Zikova *et al*., 2009), including the a-subunit detected by MS later (Skodova-Sverakova *et al*., 2015). Characterization of the trypanosomal F_1_-ATPase released by chloroform from mitochondrial membrane fragments and purified by two-step chromatography (Gahura and Zikova, 2019) showed that the catalytic subcomplex contains three copies of the euglenozoan-specific p18-subunit in addition to the universally conserved subunits *α*, *β*, *γ*, *δ* and *ɛ* (Gahura *et al*., 2018*b*). The F_1_-ATPase structure determined by X-ray crystallography further revealed that each copy of p18-subunit, an *α*-helical protein with three pentatricopeptide repeats, associates peripherally with one of the three *α* chains (Fig. 3A), and does not contribute directly to the catalytic mechanism (Montgomery *et al*., 2018). Although the atomic structure did not explain the function of p18, the elaboration of the F_1_ head in Euglenozoa by this additional subunit is extraordinary, because the *α*_3_*β*_3_*γ* subcomplex is invariant in all other known F-type ATP synthases. In addition, the *α*-subunit was found to be proteolytically cleaved at two sites separated by eight amino acid residues, producing two fragments *α*_1–127_ and *α*_135–560_, both stably associated with the complex. The split of *α*-subunit into two parts was also demonstrated in other euglenozoan protists *Crithidia fasciculata* (Speijer *et al*., 1997), *Leishmania tarentolae* (Nelson *et al*., 2004) and *Euglena gracilis* (Sathish Yadav *et al*., 2017). The structure of the trypanosomal F_1_-ATPase showed that the cleavage occurs at the region corresponding to a loop on the surface of F_1_-ATPases from other organisms and splits the N-terminal *β*-barrel domain from the rest of the protein (Montgomery *et al*., 2018; Fig. 3A and B). Nevertheless, the architecture of the entire (*αβ*)_3_-headpiece, including the nucleotide binding pockets, is highly similar to the prototypical bovine F_1_-ATPase (Bowler *et al*., 2007) and the biological relevance of the *α*-subunit cleavage remains unclear.

So far, there is no available structure of a complete ATP synthase from any trypanosomatid species. However, low-resolution structures obtained by subtomogram averaging documented general morphological similarity between ATP synthase dimers from *T. brucei* and *E. gracilis*, a representative of euglenids, a lineage closely related to kinetoplastids (Muhleip *et al*., 2017). An atomic model of dimeric ATP synthase from *E. gracilis* has recently been determined by cryo-EM (Muhleip *et al*., 2019). Apart from the universally conserved components of mitochondrial ATP synthases (*α*, *β*, *γ*, *δ*, *ɛ*, a, c and OSCP), the *E. gracilis* enzyme contains seven subunits with previously recognized homology (Sathish Yadav *et al*., 2017) to proteins found in the complex from *T. brucei* (Zikova *et al*., 2009). To get a deeper insight into the trypanosomal ATP synthase structure, we performed systematic pairwise sequence comparison of all remaining subunits in the complex from *E. gracilis* with all non-conserved trypanosomal ATP synthase components, and propose homology of four additional protein pairs, which exhibit >20% sequence identity (Fig. 3C). Overall, 19 of 23 subunits of the trypanosomal ATP synthase have homologs found in the structure from *E. gracilis*. Taken together, although the ATP synthases from *E. gracilis* and *T. brucei* are not compositionally identical, it is conceivable to assume that the structural features shaped by the conserved subunits are shared between the two species (Fig. 3C), and possibly among Euglenozoa in general.

The peripheral stalk in euglenozoan ATP synthases differs markedly from other lineages. The universally conserved OSCP features a long and partially flexible C-terminal extension, which bridges the core of the protein on the top of F_1_ with ATPTB2, a highly divergent and extended homolog of the d-subunit from opisthokontal ATP synthases anchoring the peripheral stalk to the membrane. The interaction of ATPTB2 with the extension of OSCP is clamped between two laterally positioned lineage-specific globular proteins ATPTB3 and ATPTB4 on one side and one copy of p18 on the other side. The contacts of the peripheral stalk with p18 contribute to the immobilization of the (*αβ*)_3_-hexamer (Fig. 3D). However, the reinforcement of the interaction between the F_1_ head and the peripheral stalk is unlikely the only role of p18, because the protein is essential for the integrity of the F_1_-ATPase (Gahura *et al*., 2018*b*), in contrast to the peripheral stalk components OSCP (Hierro-Yap *et al*., 2021) and ATPTB2/d-subunit (Subrtova *et al*., 2015). In *E. gracilis*, the extensions of OSCP and d-subunit compensate for the reduction of the b-subunit, the major constituent of the peripheral stalk in bacteria and mitochondria of mammals and yeast. Sequence comparison did not reveal a homolog of the b-subunit in the ATP synthase of *T. brucei*, suggesting that it might be further reduced or completely absent in Trypanosomatida.

In all reported F-type ATP synthases, the proton channel-forming a-subunit contains two horizontal membrane-embedded helices H5 and H6, which are directly involved in proton translocation (Guo *et al*., 2017; Murphy *et al*., 2019). In mammals (Spikes *et al*., 2020) and yeast (Guo *et al*., 2017) the two helices are lined by a series of six parallel transmembrane helices, contributed by one helix of each of subunits b, f, 8, i/j and k, and by a transmembrane helix of the a-subunit itself. The same configuration is observed in *E. gracilis* (Fig. 3E), suggesting the conservancy of the contributing subunits. Although the ATP synthase in *T. brucei* contains homologs of *E. gracilis* subunits k and i/j, the homologs of subunits b, f and 8 were not identified. The dimer interface of the ATP synthase in *E. gracilis* is formed largely by subunits ATPEG1 and ATPEG2 without discernible homology outside Euglenozoa, but with apparent homologs in Trypanosomatida (called ATPTB9 and ATPTB8 in *T. brucei*; Perez *et al*., 2014; Fig. 3C and F). The presence of these two proteins in *T. brucei* together with the fact that the dimers from both species arrange into short left-handed helices, as showed by cryo-electron tomography (cryo-ET; Muhleip *et al*., 2017), strongly suggests that the dimerization is mediated by the same elements. The dimer in *E. gracilis* is further stabilized by a homotypic interaction of extensions of d-subunit adopting a ferredoxin-like fold on the matrix side of the membrane. This extension is specific to euglenids and the interaction is therefore completely missing in *T. brucei*, possibly explaining the poor stability of its ATP synthase dimers (our observation). Notably, the dimer interface in *Euglena* contains several ordered phospholipid molecules, predominantly cardiolipins (Muhleip *et al*., 2019; Fig. 3F). Molecules of cardiolipin are presumably integrated also in the trypanosomal ATP synthase, as cardiolipin plays a vital role in the stability of the complex (Serricchio *et al*., 2020).

## Role of the ATP synthase in trypanosomatid parasites

The function of the ATP synthase in trypanosomatid parasites depends on the species and their life cycle stage. Digenetic trypanosomatid parasites (e.g. *T. brucei*, *T. cruzi* and *Leishmania*) possess a complex life cycle as they alternate between the insect vector and a mammalian host. In humans, they cause dreadful diseases (African trypanosomiasis, Chagas disease and leishmaniases). The scarcity of effective treatments and weak vaccine prospects drive an ongoing urgent need to identify new therapeutic targets. Considering the ATP synthase's central position in energy metabolism and its structural and compositional divergence from the mammalian counterpart, one may consider this complex as a promising drug target. The feasibility of targeting the ATP synthase is underscored by the recent approval of bedaquiline, a specific F-ATP synthase inhibitor that blocks the c-ring rotation, to treat multi-drug resistant tuberculosis (Preiss *et al*., 2015). Whereas the function of the ATP synthase in *T. brucei* parasites is well understood, the exact role of the ATP synthase and its requirement for virulence and survival of *Leishmania* and *T. cruzi* parasites are not well defined yet.

### Role of the ATP synthase in intracellular amastigotes of *Leishmania* and *T. cruzi* parasites

**Fig. 4.**
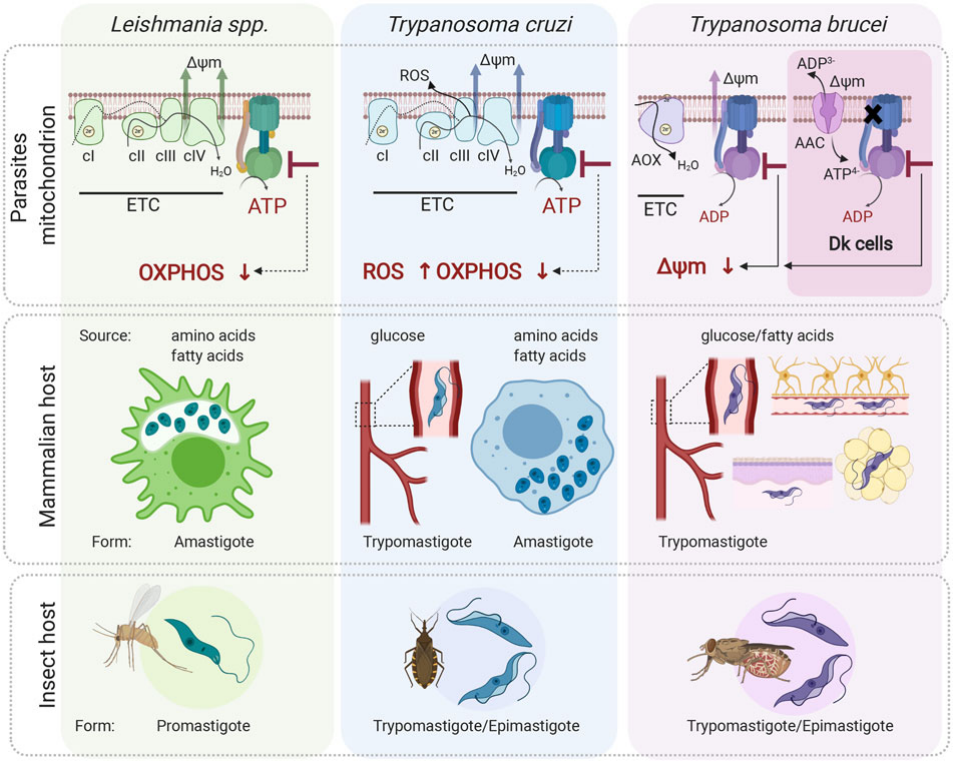
Role of the mitochondrial ATP synthase in *Leishmania*, *Trypanosoma cruzi* and *T. brucei* disease-causing life cycle stages. In the mammalian host, *Leishmania* parasites proliferate in macrophages as intracellular amastigotes, while *T. cruzi* parasites exist in two different forms; as intracellular amastigotes of various mammalian cells and as extracellular trypomastigotes in the host bloodstream. In these life forms, ATP synthase is involved in oxidative phosphorylation and generates ATP. Its inhibition is predicted to cause a depletion of cellular ATP levels. Interestingly, in *T. cruzi* trypomastigotes, the ATP synthase inhibition would also lead to increased levels of ROS due to differential expression of ETC complexes III and IV. *Trypanosoma brucei* parasites proliferate in the mammalian bloodstream but also invade interstitial spaces of various tissues such as brain, adipose tissue and skin. Importantly, *T. brucei* ATP synthase works in reverse and maintains mitochondrial membrane potential (Δ*ψ*_m_) even in trypanosomes lacking mitochondrial DNA. These Dk parasites employ vestigial ATP synthase coupled to ATP/ADP carrier (AAC) to generate Δ*ψ*_m_ electrogenically. Inhibition of F-ATPase in both forms leads to dissipation of Δ*ψ*_m_ and cell death. All three aforementioned parasites (except for Dk *T. brucei*) possess a digenetic life cycle involving insect vectors, namely sand fly for *Leishmania*, triatomine bug for *T. cruzi* and tsetse fly for *T. brucei* transmission. Figure created with Biorender.com.

*Leishmania* parasites infect a mammalian host when an infected sand fly takes a blood meal. The promastigote form is released to the skin and is rapidly internalized by macrophages. Inside the macrophages, promastigotes differentiate to aflagellated and non-motile amastigotes, which multiply in the acidic environment of the phagolysosome-like parasitophorous vacuole, whose exact metabolic content has not been determined yet. The environment most likely offers a wide range of carbon sources (sugars, amino acids and fatty acids) for the parasite to salvage due to macrophage polarization to the M2 state, which exhibits increased oxidative metabolism and promotes *Leishmania* parasites growth (Saunders and McConville, 2020). Despite this seemingly plentiful environment, intracellular amastigotes do not proliferate rapidly (Kloehn *et al*., 2015), but rather activate a stringent metabolic response, a protective mechanism to confer resistance to multiple cellular stresses (oxidative, nutritional and pH). This programmed response is associated with reduced rates of glucose uptake and with low metabolic activity to possibly minimize reactive oxygen species (ROS)-sensitive processes and to lessen the levels of endogenously generated ROS (McConville *et al*., 2015). The glucose-sparing metabolism of the intracellular amastigotes questions the importance of oxidative phosphorylation, and hence ATP production by the ATP synthase, for the parasite. Nevertheless, amastigotes show increased reliance on mitochondrial metabolism, including tricarboxylic acid (TCA) cycle and fatty acid *β*-oxidation (Saunders *et al*., 2014). These pathways generate reduced electron carriers NADH and FADH_2_ that are reoxidized by the ETC coupled to ATP synthase (Dey *et al*., 2010; Saunders *et al*., 2014). The ablation of aconitase, a TCA cycle enzyme, cytochrome *c* oxidase, or ATP synthase is predicted to be lethal by an *in silico*-generated model of the metabolic network (Subramanian *et al*., 2015). Indeed, the inhibition of ATP synthase by the cell-permeable antimicrobial peptide histatin 5 arrests of *Leishmania* amastigotes' growth (Luque-Ortega *et al*., 2008). Nevertheless, to prove that oxidative phosphorylation is critical for the *Leishmania* amastigote forms, further studies are necessary (Fig. 4).

*Trypanosoma cruzi* parasites are transmitted to humans by a blood-sucking triatomine bug through contact with its infected feces. In the skin wound, metacyclic trypomastigotes invade various types of cells, escape the lysosome-derived parasitophorous vacuole and establish infection in the host cell cytoplasm as intracellular amastigotes (de Souza *et al*., 2010). During *T. cruzi* trypomastigotes' transition to amastigotes, there is a dramatic shift from sugar-based metabolism to catabolism of amino and fatty acids manifested by repression of glucose transporters and increased levels of TCA cycle enzymes and proteins involved in fatty acid oxidation and oxidative phosphorylation. The oxidative metabolism of amino and fatty acids suggests a crucial role for oxidative phosphorylation to generate ATP (Atwood *et al*., 2005; Silber *et al*., 2009; Li *et al*., 2016; Shah-Simpson *et al*., 2016). This is further supported by the identification of highly selective compounds (i.e. GNF7686 and ELQ271) that target complex III and inhibit the growth of intracellular amastigotes in a dose-dependent manner (Khare *et al*., 2015).

After several rounds of intracellular replication, amastigotes differentiate to trypomastigotes, which are released from the cell by its rupture. Extracellular trypomastigotes disseminate and infect other cells or they are ingested by the triatomine bug to conclude the life cycle. Interestingly, the extracellular bloodstream trypomastigotes switch back to glycolysis to meet their energy demands but still employ ETC complexes to maintain the mitochondrial membrane potential. Moreover, disproportional changes in expression of ETC complexes II, III and IV create a bottleneck forcing electrons to prematurely reduce molecules of oxygen and generate significant levels of ROS (Goncalves *et al*., 2011). This induced production of ROS probably provides a redox-mediated pre-conditioning to evoke protection against host-induced oxidative challenge once the parasite enters the cell again. In this particular case, the ETC linked to ATP synthase also serves as an adaptive process allowing parasites to survive redox challenges imposed by the host. Presumably, inhibition of the ATP synthase would cause a decrease in ATP levels, which by itself can be lethal, but it would also further increase the already elevated ROS levels. Although there is evidence that the oxidative environment stimulates the parasite's growth and ROS are critical for successful infection of *T. cruzi* in various cell types (Paiva and Bozza, 2014), very high levels of ROS reverse this favourable effect and are harmful for the parasite (Goes *et al*., 2016). Therefore, an additive effect of endogenous and exogenous ROS bursts may induce deleterious changes resulting in parasites' elimination by oxidative damage (Fig. 4).

Despite some evidence that mitochondrial ETC and ATP synthase might be essential entities for the clinically relevant infectious stages of *Leishmania* and *T. cruzi* parasites, the knowledge of the mitochondrial energy metabolism in these parasites remains largely incomplete.

### The F-ATPase in the bloodstream form of *T. brucei*

The disease-causing form of *T. brucei* is an extracellular parasite inhabiting various niches of its mammalian host, including the bloodstream, adipose tissue, skin or central nervous system (Silva Pereira *et al*., 2019). Since these environments offer different nutrients, the BSF mitochondrion maintains its complexity and plasticity despite its largely reduced size compared to the insect stage organelle (Zikova *et al*., 2017). Uniquely, the parasite's mitochondrion employs ATP synthase as an ATP-consuming proton pump (Fig. 4).

The discovery of the oligomycin-sensitive F-ATP synthase in BSF by Opperdoes *et al*. (1977), elicited the curiosity of scientists, who back then considered the BSF mitochondrion a vestigial organelle with no obvious role in cellular metabolism. Seeking if this ‘promitochondrion’ possesses mitochondrial membrane potential, Nolan and Voorheis reported the existence of electrochemical gradients in BSF trypanosomes, documented by the accumulation of the radiolabelled lipophilic cation methyltriphenylphosphonium. One year after, the same authors reported that oligomycin caused a collapse of the mitochondrial membrane potential identical in magnitude to that achieved by the proton uncoupler FCCP, concluding that in BSF cells, the nature of the mitochondrial electrical gradient was exclusively attributable to the proton-pumping activity, or reverse mode, of the ATP synthase (Nolan and Voorheis, 1992). In more than 10 years, RNA interference silencing of two F_1_-ATPase subunits further corroborated the previous findings and confirmed the unusual and essential function of this enzyme for the survival of BSF trypanosomes (Schnaufer *et al*., 2005; Brown *et al*., 2006).

The role of the F-ATPase in BSF *T. brucei* strikingly contrasts with that of other eukaryotes, where the reverse operation of the ATP synthase is a rare and short-term phenomenon associated with hypoxia or anoxia. For example, under ischaemic conditions in mammalian tissues, respiration is halted and the ATP synthase reverses in response to decreased mitochondrial membrane potential, partially compensating for its loss by utilizing ATP produced by glycolysis. To limit the detrimental depletion of cellular ATP caused by an ATP-consuming F-ATP synthase (Rouslin *et al*., 1986; Jennings *et al*., 1991; St-Pierre *et al*., 2000), the activity of this enzyme is regulated by a short naturally occurring protein, the inhibitory factor 1 (IF1) (Pullman and Monroy, 1963; Walker, 1994). IF1 specifically inhibits the ATPase activity with no effect on ATP synthesis (Pullman and Monroy, 1963; Asami *et al*., 1970), although some authors propose an inhibitory effect of IF1 on both ATP synthase modes (Harris *et al*., 1979; Schwerzmann and Pedersen, 1981; Formentini *et al*., 2012; Garcia-Bermudez and Cuezva, 2016). IF1 has also been implicated in cristae biogenesis, dimerization of the ATP synthase and metabolic reprogramming of cancer cells (Campanella *et al*., 2009). This regulatory protein has been identified in *T. brucei* (TbIF1) and related trypanosomatid parasites. In *T. brucei* the expression of TbIF1 is tightly regulated throughout the life cycle as it is expressed only in the insect stages, but not in BSF (Panicucci *et al*., 2017). In *T. cruzi* and *Leishmania* parasites, regulation of IF1 expression is reflected in its slight upregulation in the mammalian intracellular stages compared to the respective insect stages in agreement with increased mitochondrial activity of *T. cruzi* and *Leishmania* amastigotes (Li *et al*., 2016; Inbar *et al*., 2017). As in other organisms, TbIF1 inhibits the hydrolytic but not the synthetic activity of the mitochondrial ATP synthase *in vitro* (Panicucci *et al*., 2017; Gahura *et al*., 2018*a*). The artificial expression of TbIF1 is lethal for BSF trypanosomes, due to the abolishment of the mitochondrial membrane potential (Panicucci *et al*., 2017).

In addition to its role in the maintenance of mitochondrial membrane potential, a functional F-ATPase is also required by BSF parasites to prevent the intramitochondrial accumulation of ATP. Elevated ATP levels lead to inhibition of the alternative oxidase, the only terminal oxidase in BSF parasites, and consequently to a reduced respiration rate (Luevano-Martinez *et al*., 2020). This represents a novel and unique role of the F-ATPase connecting this enzyme to the regulation of the glycolytic pathway, which is intimately linked to respiration *via* the alternative oxidase (Bakker *et al*., 1999). Despite these fundamental roles, BSF trypanosomes can withstand a loss of approximately 90% of the membrane-bound F-ATPase complexes with no obvious deleterious effect on their viability in culture nor on the mitochondrial membrane potential in live cells (Hierro-Yap *et al*., 2021). We speculate that the long-term tolerance of reduced levels of F-ATPase complexes might be favourable for the emergence of trypanosomes lacking mitochondrial genome, as it might provide the time frame for the parasites to gain key nuclear mutation(s) in the F-ATPase that enable(s) generation of mitochondrial membrane potential in the absence of mitochondrial-encoded a-subunit.

### The vestigial F-ATPase in trypanosomes without mitochondrial genome

The mitochondrial DNA of trypanosomatids, which is arranged in a distinct submitochondrial structure termed kinetoplast (hence kinetoplast DNA, kDNA), encodes mostly for the components of the oxidative phosphorylation pathway, key for the survival of *T. brucei*'s insect stages (Stuart, 1983; Schnaufer *et al*., 2002). In BSF trypanosomes, which do not carry out oxidative phosphorylation, kDNA is indispensable due to the requirement of the F-ATP synthase a-subunit involved in proton translocation across the inner mitochondrial membrane. To synthesize the a-subunit, BSF mitochondria express additional two kDNA-encoded proteins, uS3m and uS12m, core components of mitochondrial ribosomes (Ramrath *et al*., 2018). However, there are BSF trypanosomes that partially or completely lack their kDNA and they are referred to as dyskinetoplastic (Dk) subspecies (Agbe and Yielding, 1995). The Dk trypanosomes thriving in nature, *T. brucei evansi* (Hoare, 1937) and *T. brucei equiperdum* (Tobie, 1951), cause surra and dourine, respectively, devastating diseases that affect a broad range of domestic and wild animals in Africa, Asia and South America (Brun *et al*., 1998). Stable kDNA-deficient strains can also be generated artificially using compounds targeting the kDNA network, such as ethidium bromide or acriflavine (Stuart, 1971; Riou *et al*., 1980; Riou and Benard, 1980). Although Dk trypanosomes were thought to be locked in the long slender BSF stage, the absence of kDNA does not impair their differentiation into the stumpy form, a transmission pre-adapted stage with partially activated mitochondrial functions. However, the absence of kDNA does reduce the lifespan of the stumpy form (Dewar *et al*., 2018), hampering their differentiation into the procyclic form (Timms *et al*., 2002). Therefore, Dk trypanosomes are transmitted between hosts during sexual intercourse or mechanically *via* blood-sucking insects or vampire bats (Brun *et al*., 1998), which facilitated their spread outside the area of the tsetse fly belt (Lun and Desser, 1995).

Because the ATP synthase a-subunit is absent in Dk trypanosomes, the molecular mechanism for the generation of mitochondrial membrane potential is analogous to that of mammalian *ρ*^0^ cells and yeast *petite* mutants, both of which lack mitochondrial genome. The mechanism involves ATP hydrolysis by the vestigial ATP synthase, which provides the substrate for the electrogenic exchange of mitochondrial ADP^3−^ for cytosolic ATP^4−^ by the ATP/ADP carrier (AAC). Accordingly, oligomycin, an inhibitor that blocks the c-ring rotation, has virtually no effect on the growth of *T. b. evansi* (Schnaufer *et al*., 2005). Moreover, Dk strains contain one of several point mutations in the nuclear-encoded subunits of the F_1_-ATPase, most often the L262P or A273P substitutions in the *γ*-subunit (Lai *et al*., 2008; Dean *et al*., 2013). These substitutions are sufficient to allow the loss of kDNA and enable the generation of mitochondrial membrane potential in an F_0_-independent manner, perhaps by enhancing the hydrolytic activity of the F_1_-ATPase (Dean *et al*., 2013).

The functional independence from the F_0_ section led to the long-standing belief that the F_1_ moiety exists as a soluble entity detached from the inner mitochondrial membrane in Dk trypanosomes (Schnaufer *et al*., 2005; Jensen *et al*., 2008). However, the finding that the silenced expression of the peripheral stalk subunit ATPTB2/d-subunit slowed the growth of Dk trypanosomes *in vitro* (Subrtova *et al*., 2015) indicates that the complex might be attached to the membrane. Considering the presence of a membrane-bound F-ATP synthase in cells devoid of mitochondrial genome in other organisms (Wittig *et al*., 2010; He *et al*., 2017), it is likely that the vestigial F-ATPase in Dk trypanosomes also remains associated with the membrane to increase the spatial proximity between the AAC and the F_1_-ATPase, and ensure an efficient exchange of substrates between the two entities.

## Perspectives

Although many architectural features of ATP synthase from *Euglena* can be extrapolated to Trypanosomatida, a high-resolution cryo-EM structure of a trypanosomatid ATP synthase will be necessary for an unbiased and complete description of the enzyme in this parasitic lineage. Such research will reveal structural divergency, predominantly in the membrane-embedded part of the enzyme, as suggested by the absence of apparent homologs of some conserved ATP synthase subunits in *T. brucei* (Fig. 1). If several rotational states of the enzyme are characterized, the study would explain how the apparently flexible peripheral stalk immobilizes the catalytic subcomplex and what is the contribution of its unprecedented contacts with the F_1_-ATPase (Fig. 3D). An atomic model of trypanosomal ATP synthase could also reveal details with potential relevance to structure-based drug development. For example, the IF1-F_1_-ATPase interaction interface can be exploited by peptidomimetics to develop inhibitors selective for ATP hydrolytic activity of the enzyme. In the insect stage of *T. brucei*, the dimers of ATP synthase contribute to the shaping of the inner mitochondrial membrane by assembling into short helices on the rims of discoidal cristae (Muhleip *et al*., 2017). However, it is not clear how the dimers arrange in the BSF cells, which exhibit reduced complexity of mitochondrial ultrastructure. The understanding of the molecular mechanisms of membrane reshaping during the *T. brucei* life cycle, which can now be mimicked *in vitro* (Kolev *et al*., 2012; Dolezelova *et al*., 2020), will further illuminate the principles underlying mitochondrial dynamics in eukaryotes in general. Furthermore, recent data suggested interaction of the ATP synthase complex with mitochondrial calcium uniporter complex, AAC and phosphate carrier (Huang and Docampo, 2020). Further studies scrutinizing this putative megacomplex may reveal ATP synthase regulation by Ca^2+^ and strengthen the importance of the functional interplay between ATP synthase and both carriers in BSF and Dk cells. Considering our detailed knowledge of ATP synthase's role in *T. brucei* parasites, it is surprising that very little is known about its function in medically important parasites, *Leishmania* and *T. cruzi*. Although this gap is caused most likely by the fact that these parasites are less amenable to genetic manipulation, this can now be overcome by using subgenomic CRISPR/Cas9 libraries to dissect the role of oxidative phosphorylation complexes in different life cycle forms of these parasites. This will lead to a better understanding of the parasites' metabolic flexibility and capacity, ultimately advancing our efforts to target mitochondrial essential metabolic pathways for drug development.
